# Effect of osteopathic techniques on human resting muscle tone in healthy subjects using myotonometry: a factorial randomized trial

**DOI:** 10.1038/s41598-022-20452-9

**Published:** 2022-10-10

**Authors:** Lucas Bohlen, Jonah Schwarze, Jannik Richter, Bernadette Gietl, Christian Lazarov, Anna Kopyakova, Andreas Brandl, Tobias Schmidt

**Affiliations:** 1 Osteopathic Research Institute, Osteopathie Schule Deutschland, Hamburg, Germany; 2 Osteopathie Schule Deutschland, Hamburg, Germany; 3 Dresden International University, Dresden, Germany; 4 Erasmus School of Economics, Erasmus University Rotterdam, Rotterdam, The Netherlands; 5 Institute of Interdisciplinary Exercise Science and Sports Medicine, MSH Medical School Hamburg, Hamburg, Germany

**Keywords:** Health care, Medical research

## Abstract

Musculoskeletal disorders (MSDs) are highly prevalent, burdensome, and putatively associated with an altered human resting muscle tone (HRMT). Osteopathic manipulative treatment (OMT) is commonly and effectively applied to treat MSDs and reputedly influences the HRMT. Arguably, OMT may modulate alterations in HRMT underlying MSDs. However, there is sparse evidence even for the effect of OMT on HRMT in healthy subjects. A 3 × 3 factorial randomised trial was performed to investigate the effect of myofascial release (MRT), muscle energy (MET), and soft tissue techniques (STT) on the HRMT of the corrugator supercilii (CS), superficial masseter (SM), and upper trapezius muscles (UT) in healthy subjects in Hamburg, Germany. Participants were randomised into three groups (1:1:1 allocation ratio) receiving treatment, according to different muscle-technique pairings, over the course of three sessions with one-week washout periods. We assessed the effect of osteopathic techniques on muscle tone (F), biomechanical (S, D), and viscoelastic properties (R, C) from baseline to follow-up (primary objective) and tested if specific muscle-technique pairs modulate the effect pre- to post-intervention (secondary objective) using the MyotonPRO (at rest). Ancillary, we investigate if these putative effects may differ between the sexes. Data were analysed using descriptive (mean, standard deviation, and quantiles) and inductive statistics (Bayesian ANOVA). 59 healthy participants were randomised into three groups and two subjects dropped out from one group (n = 20; n = 20; n = 19–2). The CS produced frequent measurement errors and was excluded from analysis. OMT significantly changed F (−0.163 [0.060]; p = 0.008), S (−3.060 [1.563]; p = 0.048), R (0.594 [0.141]; p < 0.001), and C (0.038 [0.017]; p = 0.028) but not D (0.011 [0.017]; p = 0.527). The effect was not significantly modulated by muscle-technique pairings (p > 0.05). Subgroup analysis revealed a significant sex-specific difference for F from baseline to follow-up. No adverse events were reported. OMT modified the HRMT in healthy subjects which may inform future research on MSDs. In detail, MRT, MET, and STT reduced the muscle tone (F), decreased biomechanical (S not D), and increased viscoelastic properties (R and C) of the SM and UT (CS was not measurable). However, the effect on HRMT was not modulated by muscle–technique interaction and showed sex-specific differences only for F.

*Trial registration* German Clinical Trial Register (DRKS00020393).

## Introduction

Globally, musculoskeletal disorders (MSDs) accounted for ~ 1.3 billion prevalent and ~ 334.7 million incident cases in 2017^1^. Notably, most of the prevalence and incidence are attributable to gout, rheumatoid arthritis, osteoarthritis, neck pain (NP), and low back pain (LBP)^2,3^. In 2017, MSDs were the main contributor to global disability and LBP was the leading cause of disability since 1990^4^. Similarly, the global costs of MSDs due to health expenditure and production loss are reported to be immense^5^. However, these high health costs mismatch with low research investments^6^, and policy responses are thus required to close the gap^7^. Hence, MSDs are highly prevalent, burdensome, and costly.

Manual therapy seems to benefit patients with MSDs but is merely endorsed as an adjuvant treatment^8^ due to limited high-quality evidence^9^. Still, among the non-surgical and non-pharmacological interventions preferred by patients with LBP^10,11^, manual therapy provides the best evidence for an immediate-term reduction of pain and disability^12^. Thus, patients with MSDs may consult an osteopath in primary care (depending on varying country regulations and professional recognitions across the world)^9,13^.

Osteopathy is a person-centered approach to healthcare^13^ deploying both manual and patient management approaches (ranging from touch and manipulation to psychological support and lifestyle advice)^14^. Manual findings are treated using osteopathic manipulative treatment (OMT)^15^, which is primarily, but not exclusively, applied to treat MSDs like back pain conditions^16,17^. To date, there is promising evidence that OMT could be an effective and safe treatment for patients with MSDs^18^, particularly for improving pain and function in patients with spinal complaints^19^ like NP^20^ and LBP^21–24^. Hence, OMT was recommended for patients with LBP^15,25^ and was even reported to be dominant and cost-effective compared to usual care in the management of LBP and NP, respectively^26^. Still, the current body of evidence lacks robustness due to methodological shortfalls and counterevidence is available as well^27–29^.

MSDs and OMT are complex health conditions and interventions, respectively. Both are underpinned by poorly understood mechanisms^30,31^ and are associated with various biological, psychological, and social factors^32,33^. Another commonality is that MSDs^34,35^ and OMT^36–39^ are reputed to be associated with changes in muscle tone. On the one hand, alterations in lumbar myofascial tone and stiffness seem to contribute to the development and symptoms of LBP^34,35,40^. On the other hand, OMT may alter muscle tone and stiffness in patients with MSDs^36–39^. Hence, we hypothesise that a putative mechanism of action underpinning the treatment of MSDs with OMT might be the modulation of muscle tone.

However, not every technique and muscle may be relevant in the context of MSDs. On the one hand, manual techniques should be assessed that were shown to improve pain and function in patients with MSDs (e.g., LBP), which includes myofascial release techniques (MRT), muscle energy techniques (MET), and soft tissue techniques (STT)^41–49^. On the other hand, muscles should be tested that have demonstrated elevated muscle stiffness (or hyperactivity) in patients with MSDs (e.g., NP, temporomandibular disorder, and tension-type headache, but also migraine headaches), which includes the corrugator supercilii muscles (CS), superficial masseter muscles (SM), and upper trapezius muscles (UT)^50,51^.

Muscle tone is defined as the resting tension of the tissue in response to stretch, which comprises active (i.e., electrical activity within muscle cells) and passive muscle tone (intrinsic biomechanical and viscoelastic properties of the muscle)^35,52^. Moreover, the human resting muscle tone (HRMT) describes the resting tension of the whole myofascial continuum (a biotensegrity system that includes muscle and connective tissues)^53^. Although not conclusively determined^54^, the HRMT may arise due to slowly cycling cross-bridges between myosin heads and actin filaments^40,53,55^.

Previous work suggested that palpable muscle tension in patients with MSDs may reflect alterations of the HRMT^53^. Nonetheless, earlier studies on the effect of osteopathic interventions on muscle tone relied on palpation and electromyography (EMG) as measures^56–58^. However, manual palpation is reported to be unreliable^59–66^ and EMG is not informative of the HRMT^53^. Instead, an objective and reliable myotonometer should be used to assess the HRMT^34,54^. This includes the MyotonPRO, which induces oscillations in the muscle fibres as a means of quantifying biomechanical and viscoelastic muscle properties^34,35,67^. More precisely, it measures muscle tone (F [oscillation frequency]), stiffness (S [dynamic stiffness]), decrement (D [logarithmic decrement]), relaxation (R [mechanical stress relaxation time]), and creep (C [ratio of deformation and relaxation time])^68^.

Manual therapists commonly argue that treatment reduces tension and increases elasticity in muscles at rest. We hypothesize that these palpable changes may reflect a decrease in muscle tone, stiffness, and decrement as well as an increase in muscle relaxation and creep as measured with the MyotonPRO. Notably, the myometric parameter decrement (D) is inversely proportional to the muscle’s elasticity (if decrement decreases, elasticity increases)^69^. Further, we speculated that the putative effects may differ depending on which type of manual technique (e.g., MRT, MET, STT) is applied to which kind of muscle (e.g., CS, SM, UT). For example, practical experience suggests that low-pressure techniques might be preferred for smaller and thinner muscles, whereas high-pressure techniques may be favoured for larger and thicker muscles. Another factor to consider is that changes in muscle properties probably differ between sexes (although the effect seems to vary depending on the treated muscles and measured parameters)^70–75^.

Hence, in this study, we aimed to assess the effect of (OMT-related) manual techniques with different characteristics (MRT, MET, and STT) on (HRMT-related) muscle tone and biomechanical and viscoelastic muscle properties (F, S, D, R, and C) of (MSD-related) muscles with different sizes and thicknesses (CS, SM, and UT) in healthy subjects.

### Objectives

The primary objective was to evaluate the effect of MRT, MET, and STT on the HRMT (expressed by myometric parameters: F, S, D, R, and C) of the CS, SM, and UT. The effect was measured for all groups from baseline to follow-up using the MyotonPRO. We hypothesized that MRT, MET, and STT decrease F, S, and D (inverse of elasticity), and increase R and C.

The secondary objective was to evaluate if specific techniques (MRT, MET, and STT) are more effective for modulating the HRMT (expressed by myometric parameters: F, S, D, R, and C) of specific muscles (CS, SM, and UT). The effect was measured for each group from pre- to post-intervention using the MyotonPRO. We hypothesized that the predicted changes in muscle properties are preferentially achieved through MRT for the CS, MET for the SM, and STT for the UT.

The ancillary objective was to analyse if the putative effects assessed in the primary and secondary objectives differ between the sexes.

## Methods

### Trial design

This single-blinded 3 × 3 factorial randomised trial was conducted in Hamburg, Germany. No changes to the methods were made after trial commencement. The study is largely reported according to the Consolidated Standards of Reporting Trials (CONSORT) statement^76,77^ since there are currently no specific guidelines available for randomised trials using a factorial design^78^.

### Trial procedure

Demographic data was collected one month prior to the trial (t0). Participants were randomly allocated into three groups (G1, G2, and G3) undergoing three treatment sessions (t1-t3). The trial comprised one-week washout periods between each of the sessions. A session consisted of one intervention day (lasting from approximately 9 am to 4 pm), which encompassed 5 min of measurement, followed by 5 min of treatment, and renewed 5 min of measurement per subject. Baseline and follow-up refer to the first (pre-intervention) measurement at t1 and the last (post-intervention) measurement at t3, respectively.

Data were collected before and after treatment (pre- and post-intervention) in each of the three sessions. Overall, each group was measured six times. Participants started with 5 min delay to one another to allow measurement by one assessor who was not involved with the interventions. All interventions and measurements were applied (1) to the right side of the participant’s body to ensure comparability, and (2) in relaxed supine position to maintain resting muscle state. In this trial, all groups were intervention groups that were treated at one muscle per session, while all three muscles were measured. Thus, measures from untreated muscles were used as control values (Table 1). For example, in session 1, G1 was treated (and measured) at the UT, whereas the UT was not treated (but measured) in G2 or G3. Thus, G2 and G3 provided the UT control values for G1. In other words, the control values for the treated muscle in each group were generated by the untreated muscles in the other two groups.

**Table 1:** Trial procedure.

Session and technique	Group and therapist	Muscle and condition
Session 1 with MRT	G1 with P2	CS untreated as control
		SM untreated as control
		UT treated with intervention
	G2 with P1	CS untreated as control
		SM treated with intervention
		UT untreated as control
	G3 with P3	CS treated with intervention
		SM untreated as control
		UT untreated as control
Session 2 with MET	G1 with P3	CS untreated as control
		SM treated with intervention
		UT untreated as control
	G2 with P2	CS treated with intervention
		SM untreated as control
		UT untreated as control
	G3 with P1	CS untreated as control
		SM untreated as control
		UT treated with intervention
Session 3 with STT	G1 with P1	CS treated with intervention
		SM untreated as control
		UT untreated as control
	G2 with P3	CS untreated as control
		SM untreated as control
		UT treated with intervention
	G3 with P2	CS untreated as control
		SM treated with intervention
		UT untreated as control

MRT = myofascial release technique; MET = muscle energy technique; STT = soft tissue technique; G1 = group 1; G2 = group 2; G3 = group 3; P1 = practitioner 1; P2 = practitioner 2; P3 = practitioner 3; CS = corrugator supercilii muscle; SM = superficial masseter muscle; UT = upper trapezius muscle.

During each session, each group received treatment with the same osteopathic technique but applied by another practitioner to another muscle. Over the course of the trial, all three groups were treated (1) with all three osteopathic techniques (MRT, MET, and SST); (2) at all three muscles (CS, SM, and UT); and (3) by all three practitioners (P1, P2, P3). However, the muscle-technique-practitioner combination was distinct for each group during each session (Table 1).

### Participants

Undergraduate osteopathy students were recruited from the Osteopathie Schule Deutschland in Hamburg, Germany. The sample was limited to healthy subjects from this specific setting. The eligibility criteria were specified to include participants between 18 and 50 years old and exclude participants with health complaints (particularly muscle disorders) to minimise the risk of age-^71,79^ and disease-related^80^ changes of the musculature.

### Interventions

Three manual techniques from the osteopathic field (MRT, MET, and STT)^81^ were selected and administered for ~ 5 min. These osteopathic techniques were adjusted to fit the structure and function of each muscle and were applied with the aim of modifying the HRMT in healthy participants. However, it is notable that the mechanisms of action underpinning these manual techniques are not fully understood but may involve diverse (neuro-)biological processes^46,49,82–90^. The interventions were performed by three osteopaths (1:2 female to male ratio), who showed similar characteristics in terms of age (24.28 [0.20] years) and practice experience (4.83 [0.29] years). A consensus training comprising three sessions of one hour (sixty minutes per technique) was implemented prior to the trial to ensure that all therapists applied the interventions coherently. During the first session, MRT was applied to the right CS (G3), SM (G2), and UT (G1). During the second session, MET was applied to the right CS (G2), SM (G1), and UT (G3). During the third session, STT was applied to the right CS (G1), SM (G3), and UT (G2) (Table 1). Overall, the interventions were chosen to represent the broad range of osteopathic techniques and their diverse characteristics (Box 1), whereas the muscles were chosen due to their apparent differences in size and thickness (Box 2).

#### Myofascial Release Technique (MRT)

**Figure 1:**
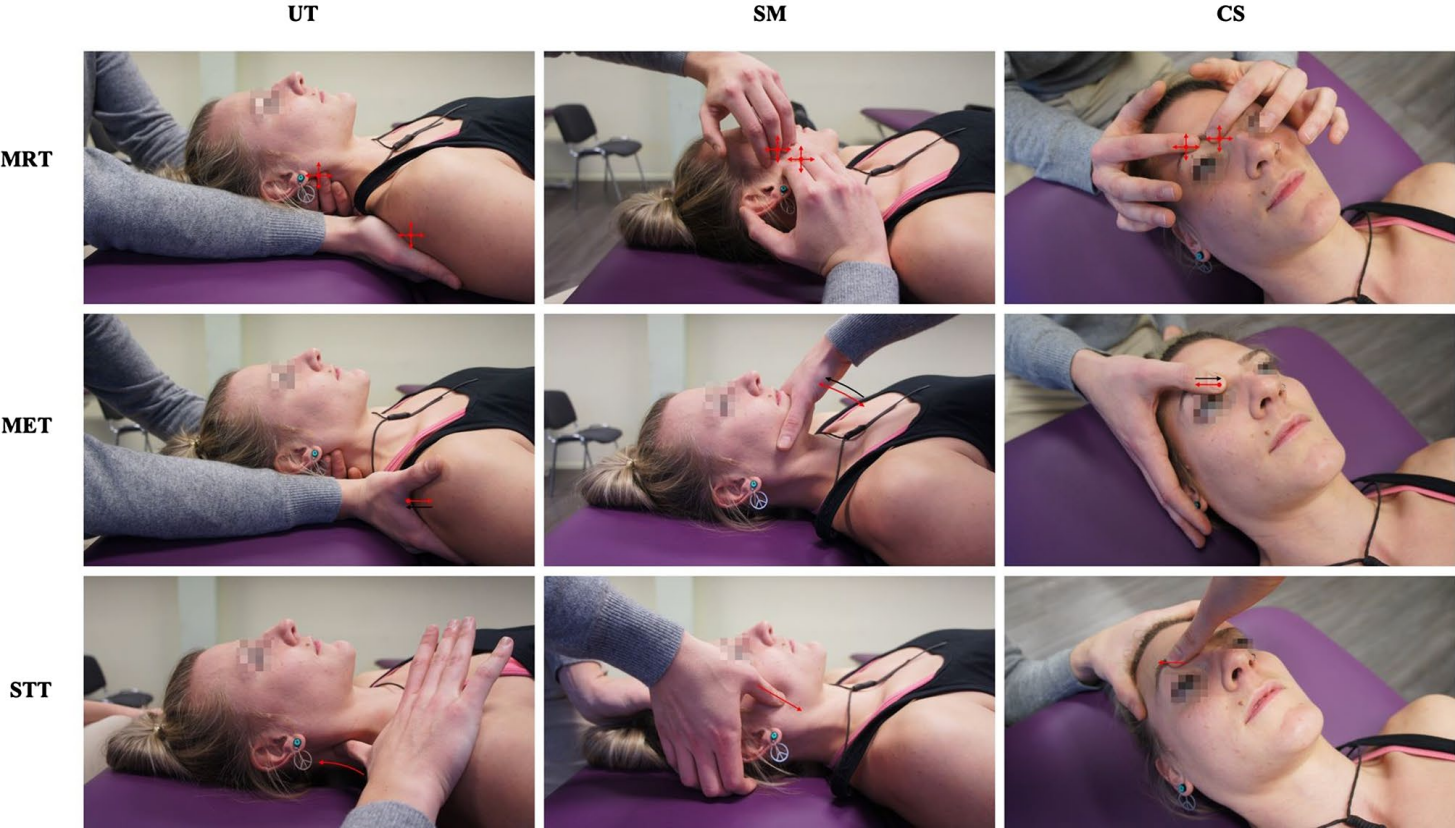
Osteopathic techniques; Legend: red arrows = therapists’ motion; black arrows = participants’ motion; four-headed arrows = motion applied in all directions; Abbreviations: MRT = myofascial release technique; MET = muscle energy technique; STT = soft tissue technique; UT = upper trapezius muscle; SM = superficial masseter muscle; CS = corrugator supercilii muscle.

MRT is an indirect (or direct) and passive technique where pressure and stretch with low load and long duration (which are adjusted based on palpatory feedback) is applied to release myofascial tissues^41,42^. The muscle is palpated (covering origin and insertion) and guided alongside the path of least resistance into a position of ease^98^, thereby following the tissues’ micro–movements away from the restricted barrier until a release occurs (Fig. 1).

#### Muscle Energy Technique (MET)

MET is a direct and active technique where the patient is instructed to voluntarily contract muscles into a controlled direction against the therapist counter-pressure^99^. In detail, the therapist brings the muscle into a position of stretch and holds it at the restriction barrier. The participant then performs an isometric contraction of the muscle (with 25% of maximum effort/force) away from this restricted barrier and against the therapist’s counterforce^100^. After approximately 3–6 s of contraction, the participant relaxes, and the therapist adjusts the tissue towards its renewed movement/restriction barrier. This post–isometric relaxation approach is repeated 3–6 times (Fig. 1).

#### Soft Tissue Technique (STT)

STT is a direct and passive technique where stretch, traction and/or deep pressure is applied to soft tissues^81^. Herein, the therapist uses repeated, slow, high and deep pressure gliding strokes applied with the thumb alongside the muscle fibres (which is similar to muscle stripping massage^101–103^) of the CS (from origin towards insertion), SM (from origin towards insertion), and UT (from insertion towards origin). This approach is similar to the treatment of a trigger band according to the fascial distortion model^104^ but is applied to palpably firm muscle fibres and their fascial surroundings (Fig. 1).

Box 1 Characteristics of the techniques Manual techniques have distinct characteristics and can roughly be differentiated as follows: (1) direct or indirect techniques: treatment is applied against or following the tissue resistance; (2) active or passive techniques: the patient is actively involved with the intervention or receives it passively; and (3) high-pressure or low-pressure techniques: manual force is applied firmly or gently^81,91^.

Box 2 Characteristics of the muscles Remarkably, the availability of data on both size and thickness of these muscles were scattered and the values given subsequently are to be interpreted with caution. On the one hand, data shows that the relaxed muscle thickness of the (1) CS ranges between 5 and 6 mm^92^; (2) SM ranges between 9 and 15 mm (notably, values account for the combined thickness of the superficial and deep part of the masseter muscle and are thus exaggerated)^93^; and (3) UT ranges between 11 and 12 mm^94^. No data was available on the surface size of these muscles. Thus, we calculated an approximate surface size based on data reporting the length and width of these muscles (which is imprecise as it does not account for factors like muscle shape). We calculated a surface size for the (1) CS of ~ 3.69 cm^2^ (length: 29.24 mm; width: 12.62 mm)^95^; (2) SM of ~ 24.40 cm^2^ (length: 6.32 cm; width: 3.86 cm)^96^; and (3) UT of ~ 540 cm^2^ (length: 45 cm; width: 12 cm) (notably, this data is based on a myocutaneous trapezius flap and likely imprecise)^97^.

### Outcomes

**Figure 2:**
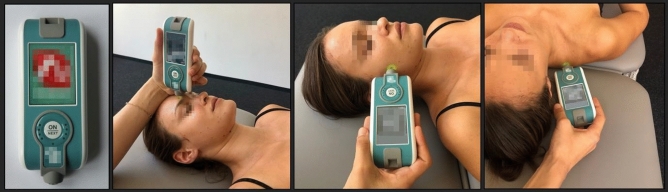
MyotonPRO; Legend: MyotonPRO and application for the CS, SM, and UT (from left to right).

We used the handheld digital palpation device MyotonPRO [Version 5.0.0] as the outcome measure (Fig. 2). This myotonometer assesses the muscle’s tone, biomechanical and viscoelastic properties using five parameters by means of dynamic oscillation mechanosignals^54,68^ (Table 2). The MyotonPRO is a valid and reliable measurement tool for healthy and diseased participants^105,106^ (Box 3) that has been applied to evaluate muscle tone, muscle stiffness, and HRMT in multiple studies investigating various structures and conditions^34,54,67,107–109^. The myotonometer measurements were carried out at all three sessions (t1-t3) before and after the treatment intervention. Measurement points (MPs) were predefined for the myotonometer measurements of each muscle prior to the trial (Box 4). MPs were identified by manual palpation following anatomical landmarks and marked before each session using a dermatological skin marker pen.

**Table 2:** MyotonPRO parameters.

Parameter	Description	Formula
F—Oscillation frequency	Measures the muscle’s tone in resting state (excluding voluntary contractions like depicted within an EMG measurement) in Hertz (Hz)	$F={f}_{max}$
S—Dynamic stiffness	Measures the biomechanical property of a muscle to deform its shape under internal or external force in Newton meter (N/m)	$S= \frac{{a}_{\mathit{max} \bullet } {m}_{probe}}{\Delta l}$
D—Logarithmic decrement	Measures the elasticity characterized by the muscle’s natural oscillation (which represents the biomechanical ability to regain its initial shape after deformation under internal or external force)	$D=l\mathrm{n}\left(\frac{{a}_{1}}{{a}_{3}}\right)$
R—Mechanical stress relaxation time	Measures the time the muscle tissue needs to recover its shape after deformation under internal or external force in milliseconds (ms)	$R={t}_{R}- {t}_{1}$
C—Ratio of deformation and relaxation time	Measures the gradual elongation of muscle tissue over time under constant tensile stress in Deborah numbers (De)	$C= \frac{R}{{t}_{1}- {t}_{T}}$

Box 3 Validity and reliability of the MyotonPRO The MyotonPRO shows good validity and high reliability for measuring, for example, the trapezius muscle^110^. In detail, studies demonstrate moderate to excellent inter-rater reliability (intra-class correlation coefficient [ICC] for F = 0.87, S = 0.79, D = 0.93, R = 0.65, C = 0.50; standard error of measurement [SEM] for F = 0.7, S = 16.8, D = 0.2, R = 1.4, C = 0.1), moderate to good intra-rater reliability (ICC for F = 0.81, S = 0.82, D = 0.76, R = 0.74, C = 0.52; SEM for F = 0.8, S = 16.9, D = 0.2, R = 1.2, C = 0.1)^111^, and good to excellent test–retest reliability (ICC for S = 0.821–0.913; SEM for S = 23.59)^112^.

Box 4 Measurement points (MPs) The MP for the: (1) CS was determined to be located 0.5 cm superior to the supraorbital notch slightly above the eyebrow^113,114^; (2) SM was determined to be located just below the midpoint of a virtual line between the muscle’s origin and attachment (masseteric tuberosity of the mandibular angle and the tendinous aponeurosis at the anterior third of the zygomatic arch)^115,116^; and (3) UT was determined to be located halfway between a virtual line from the top of the acromion to the spinous process of C7 (which is ~ 19.5 cm)^54,117^ (notably, MPs were inspired, not determined, by the cited references).

### Sample size

The sample size was calculated prospectively using G*Power, which is a power analysis for ANOVA with repeated measures (within-between interaction)^118,119^. We assumed a type I error level of 0.05 and statistical power of 95%. Based on an estimated partial η^2^ of 0.1 (unpublished data), an effect size of 0.33, and three measurements and four groups, a total sample size of 52 participants was calculated. Using an estimated drop-out rate of 15%, the sample size was planned with 60 participants.

### Randomisation

The sample was randomly allocated into three groups (G1, G2, G3) by block randomization (1:1:1 allocation ratio) using computer-generated allocation schedule (http://www.randomization.com). Furthermore, we randomly assigned which technique would be applied in which session by throwing the dice. Afterwards, we randomly assigned the muscles and therapists to the groups and session in the same manner. The principal investigator generated the random allocation sequence, enrolled participants, and assigned them to sequences of intervention while having no clinical involvement in the trial. Treatments were scheduled according to allocation sequence and therapists and participants were first introduced to each other during the respective sessions.

### Blinding

Participants and statisticians, but not therapists and assessors, were blinded to the conditions. However, we assume that blinding was compromised because the participants were osteopathy students that were likely able to distinguish between the interventions.

### Statistical methods

The outcomes from myotonometer measurements were assessed by calculating the within-participant difference of each parameter for all groups between t1 and t3 (primary objective) and the between-participant difference of each parameter for each group between pre- and post-treatment of each session (secondary objective). Statistical analysis was conducted by employing the software R Studio. Myotonometer properties (F, S, D, R, and C) were used as parameters and converted into factors. To control for intergroup comparability, myotonometer properties were tested between groups before the interventions using a one-way ANOVA. There were no outliers in the data. The variables were normally distributed as determined by the Shapiro–Wilk test (p > 0.05). Homogeneity of error variances between groups was met for all these variables according to Levene’s test (p > 0.05). The descriptive statistic for the primary objective was presented by mean, standard deviation, and quantiles, whereas the secondary objective was presented by standard deviation and quantiles. Due to the limitations of standard repeated measures ANOVA for categorical variables and unbalanced data, the inductive statistics for the primary and secondary objectives as well as subgroup analysis were calculated using the Bayesian version of the repeated-measures analysis of variance (BANOVA)^120^. Post hoc analysis was interpreted using simple effects. Heidelberger & Welch’s diagnostic was used to run length diagnostic and convergence diagnostic. The p-values of the multiple comparisons were adjusted using the Bayesian model^121^. The significance level was set to 5% (p ≤ 0.05). Missing completely at random values were included for further analysis. Missing at random and missing not at random values were excluded as they are dependent on one factor and bias the results^122^.

### Ethics approval and consent to participate

This study has obtained informed consent from participants, was carried out in accordance with the Declaration of Helsinki^123^ and was approved by the ethics committee of the Osteopathic Research Institute (Nr. 020–01). Written informed consent for publication of the images was obtained from the subjects.

## Results

**Figure 3:**
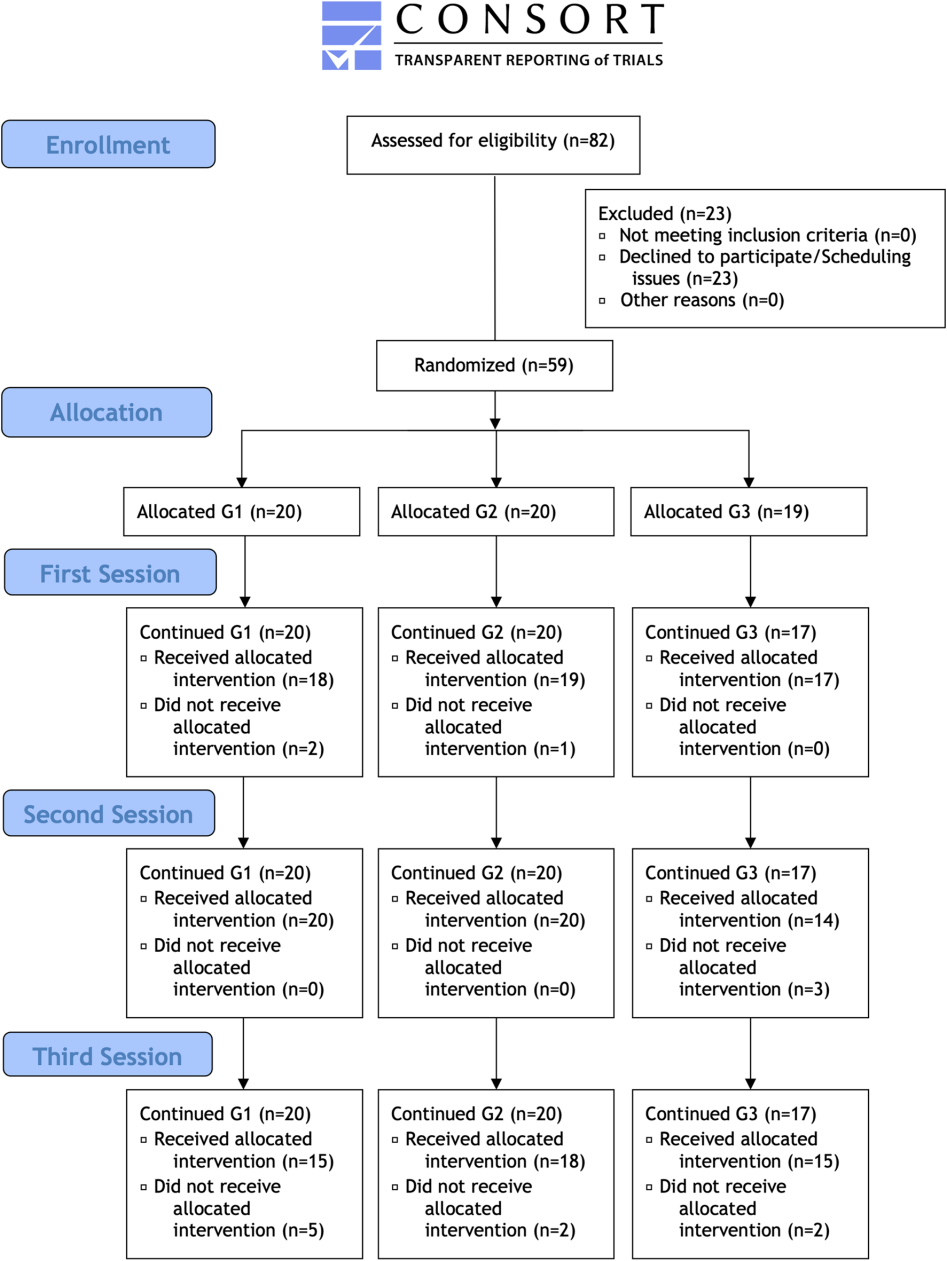
Participant flow chart; Legend: Not applicable.

Overall, 82 participants were screened and 23 declined to participate or had scheduling issues. The remaining 59 participants were randomly allocated into groups, leading to a sample of 20 participants for groups G1 and G2, and 19 participants for the G3 group. Two participants from group G3 cancelled their participation in the study due to personal reasons and were excluded from interventions and analysis prior to the first session. Therefore, the first trial session started with the final sample size (n = 57) distributed across groups as follows: G1 (n = 20), G2 (n = 20), and G3 (n = 17). Unfortunately, some participants missed scheduled appointments and thereby allocated interventions and measurements. These participants were not excluded from study participation and data was included for analysis unless all three sessions were missed, which did not occur. In detail, appointments were missed during the: (1) first session by two participants from group G1 and one participant from group G2; (2) second session by three participants from group G3; and (3) third session by five participants from group G1, two participants from group G2, and two participants from group G3 (Fig. 3).

### Recruitment

Participants were recruited, provided written informed consent, and reported demographic data in December 2019 (t0). The trial was implemented over the course of one month between January and February 2020 (t1−3).

### Demographic data

Demographics were recorded and included sex, handedness, age, and body mass index (BMI). The sample was predominantly female (68%), right-handed (91%), young (22.7 ± 4.5 years), and of normal weight (22.0 ± 2.5 BMI) (Table 3).

**Table 3:** Demographics.

Parameter	G1 (n = 20)		G2 (n = 20)		G3 (n = 17)	
	*R*	*%*	*R*	*%*	*R*	*%*
F:M	14:6	70	13:7	65	12:5	71
RH:LH	16:4	80	19:1	95	17:0	100

	Mean	SD	Mean	SD	Mean	SD
Age	22.0	1.7	21.4	2.7	25.1	7.0
BMI	21.8	1.7	21.3	2.1	23.2	3.4

G1 = group 1; G2 = group 2; G3 = group 3; F = female; M = male; RH = right-handed; LH = left-handed; BMI = body mass index; R = ratio; SD = standard deviation.

### Numbers analysed

The data were examined for availability and normality to rule out statistical errors during analysis. Overall, 8.9% of the collected data did not correspond to the confidence interval of the MyotonPRO (set to 90%) and was thus not available for analysis. All missing values arose from measurements of the CS, demonstrating that the data was not missing at random. The CS was consequently excluded from analysis because correct coefficient estimation was not guaranteed. After exclusion, data from 104 treatments (54 for SM and 50 for UT) were included for analysis (104 of 156 measures).

### Outcomes

Here, full outcome data are reported (Table 4). Subsequently, results will be presented (excluding the CS) according to the primary and secondary objectives.

**Table 4:**
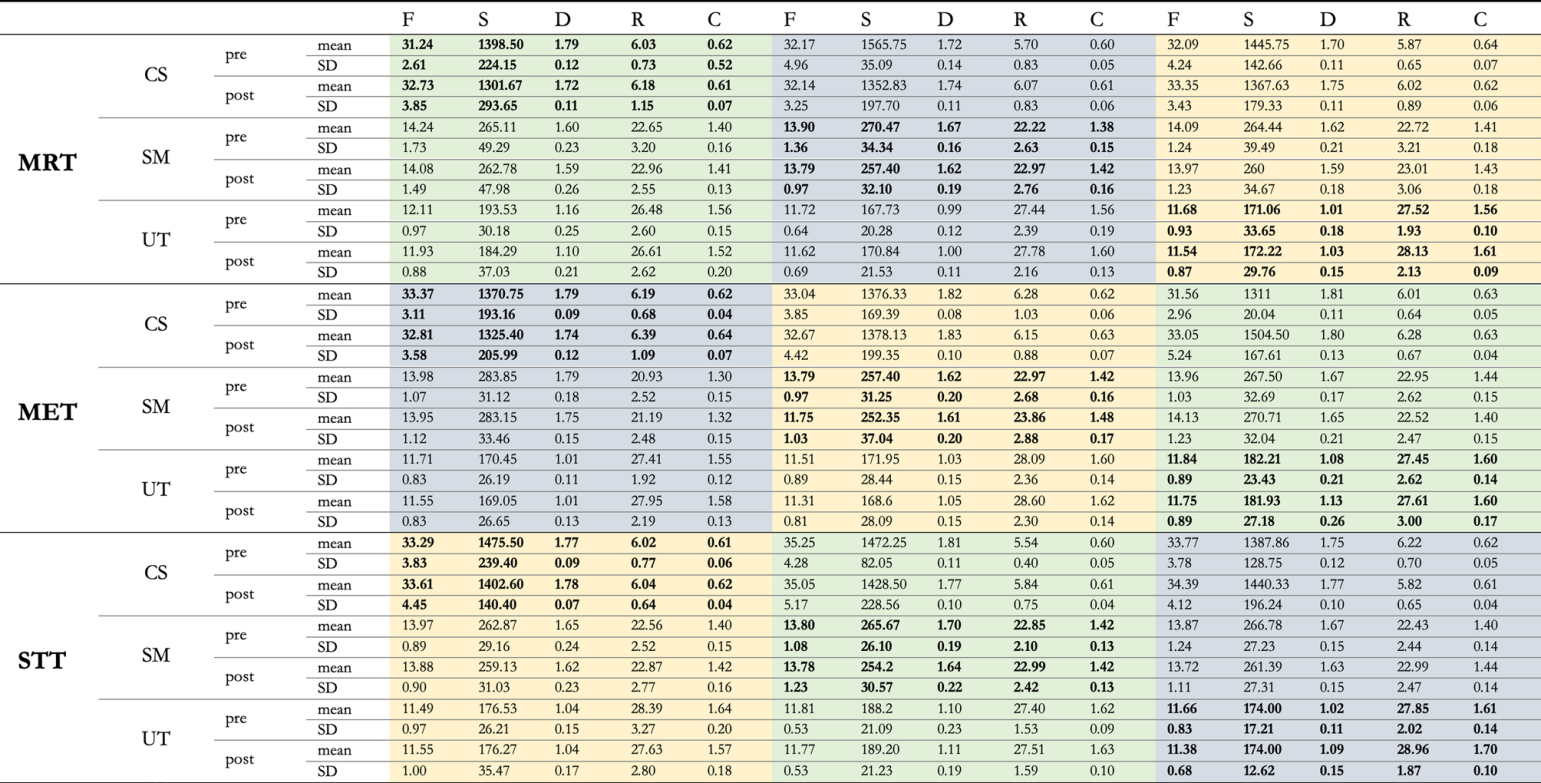
Outcomes.

Yellow = G1; blue = G2; green = G3. Bold values = treated muscles (intervention values); normal values = untreated muscles (control values). MRT = myofascial release technique; MET = muscle energy technique; STT = soft tissue technique; CS = corrugator supercilii muscle; SM = superficial masseter muscle; UT = upper trapezius muscle; pre = before intervention; post = after intervention; SD = standard deviation; F = Oscillation Frequency; S = Dynamic Stiffness; D = Logarithmic Decrement; R = Mechanical Stress Relaxation Time; C = Ratio of deformation and Relaxation time.

#### Effect of osteopathic techniques on muscle tone and biomechanical and viscoelastic properties

No significant group differences were determined by the one-way ANOVA before treatment in F (F[2,308] = 0.61; p = 0.545), S (F[2,308] = 0.94; p = 0.390), D (F[2,308] = 0.79; p = 0.454), R (F[2,308] = 1.15; p = 0.319), and C (F[2,308] = 1.63; p = 0.198). The outcomes for the primary objective were assessed by means of the standardized mean difference. The data passed all of Heidelberger's and Welch's convergence diagnoses and showed that: F (−0.163 [0.060]; p = 0.008), S (−3.060 [1.563]; p = 0.048), R (0.594 [0.141]; p < 0.001), and C (0.038 [0.017]; p = 0.028) changed significantly, while D (0.011 [0.017]; p = 0.527) did not change significantly (Table 5). In other words, muscle tone (F [p = 0.008]) and biomechanical properties (S [p = 0.048] not D [p = 0.527]) decreased, while the viscoelastic properties (R [p < 0.001] and C [p = 0.028]) increased. Subgroup analysis for sex-specific changes revealed a significant interaction for F (−0.192 [0.089]; p = 0.030), but not for S (0.008 [0.0125]; p = 0.510), D (0.008 [0.013]; p = 0.555), R (−0.423 [0.218]; p = 0.057), and C (−0.019 [0.015]; p = 0.237). Due to the significant interaction, simple effects were interpreted for sex-specific differences in muscle property changes from baseline to follow-up (Table 5).

**Table 5:** Results—changes in muscle properties for all groups from baseline to follow-up (within-participant difference).

Parameter	Sex	Mean	SD	Quantile 0.025	Quantile 0.075	p-value
**F**	total	−0.163	0.060	−0.278	−0.045	**0.008**
	female	−0.209	0.073	−0.344	−0.059	**0.003**
	male	−0.106	0.116	−0.335	0.120	0.345
**S**	total	−3.060	1.563	−6.187	−0.011	**0.048**
	female	−4.152	1.831	−7.707	−0.540	**0.023**
	male	−1.305	2.829	−6.747	4.310	0.628
**D**	total	0.011	0.017	−0.022	0.045	0.527
	female	0.018	0.012	−0.006	0.040	0.120
	male	−0.001	0.013	−0.026	0.024	0.906
**R**	total	0.594	0.141	0.311	0.873	** < 0.001**
	female	0.678	0.182	0.321	1.044	** < 0.001**
	male	0.471	0.256	−0.041	0.983	0.074
**C**	total	0.038	0.017	0.004	0.071	**0.028**
	female	0.045	0.013	0.020	0.071	** < 0.001**
	male	0.029	0.015	−0.001	0.059	0.059

F = Oscillation Frequency; S = Dynamic Stiffness; D = Logarithmic Decrement; R = Mechanical Stress Relaxation Time; C = Ratio of deformation and Relaxation time; SD = standard deviation; Bold values = p < 0.05; P-value for the simple effects.

#### Modulation of the effect of osteopathic techniques on muscle properties through muscle-technique pairing

**Figure 4:**
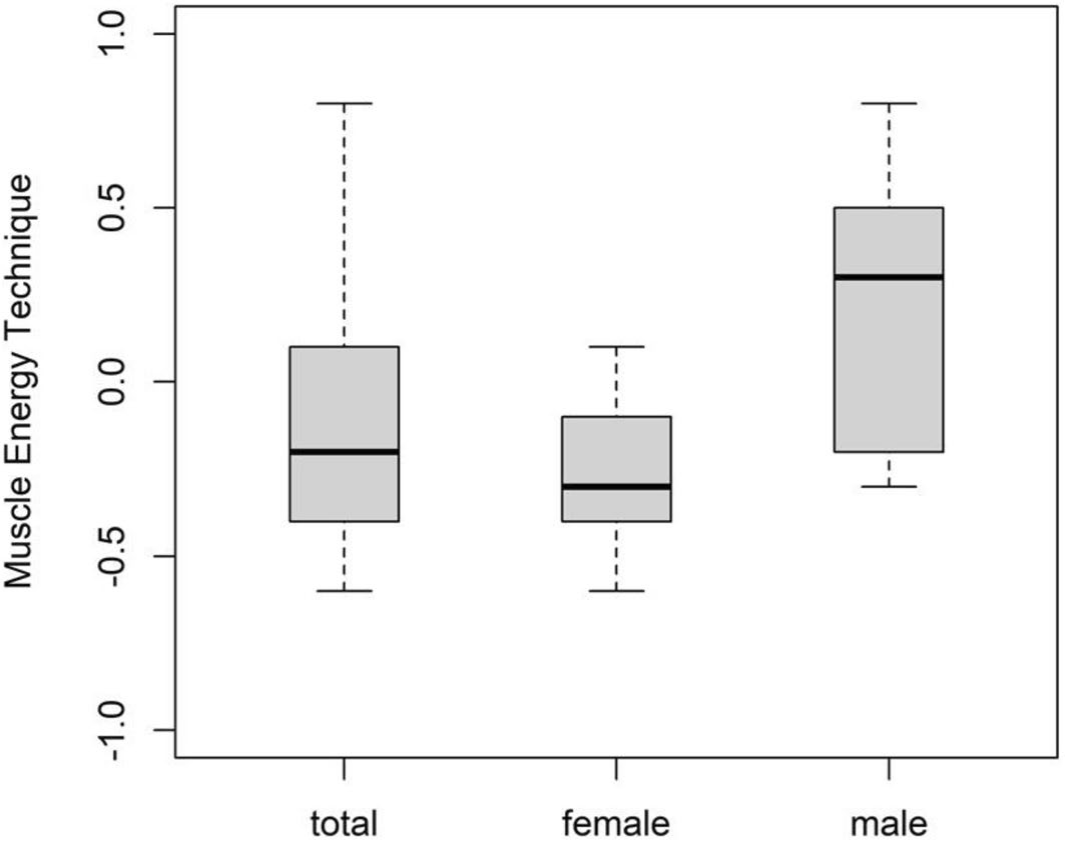
Mean differences in frequency of UT for MET; Legend: Not applicable.

**Figure 5:**
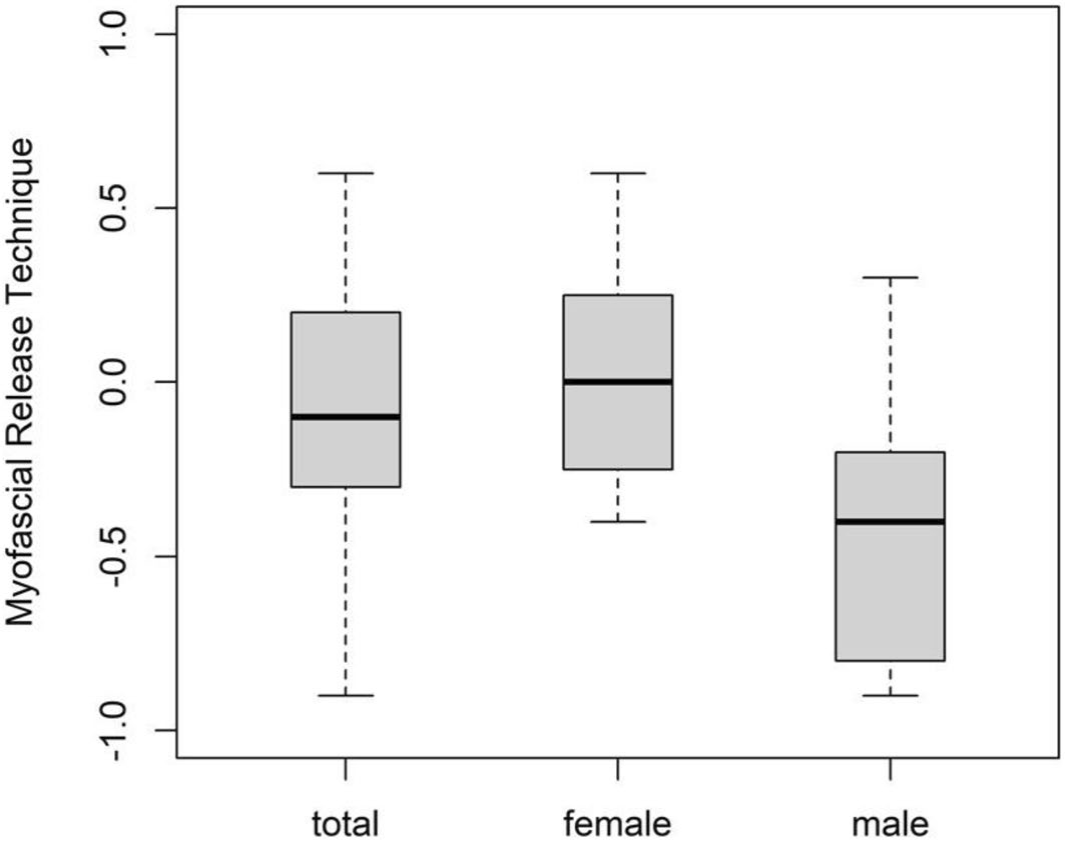
Mean differences in frequency of UT for MRT; Legend: Not applicable.

Since there was a significant interaction between treatment and muscle (0.037 [0.014]; p = 0.009), the simple effects were interpreted for the secondary objective. There was a tendency for a difference in comparison between MRI, MET and STT, but all multiple comparisons between treatment and muscle were not significant (p > 0.05) (Table 6). There was no sex-specific simple effect for the significant interaction but some tendency (p > 0.05). For example, in males compared to females, F of the UT showed a higher increase and decrease following MET and MRT, respectively (Table 7 and Figs. 4, 5).

**Table 6:** Results—changes in muscle properties for each group from pre- to post-intervention in each session (between-participant difference).

Parameter	Muscle	Treatment	Simple effect	SD	Quantile 0.025	Quantile 0.075	p-value
F	SM	MET	−0.084	0.121	−0.320	0.147	0.498
		MRT	−0.061	0.120	−0.307	0.171	0.598
		STT	0.146	0.126	−0.100	0.395	0.244
	UT	MET	0.073	0.132	−0.189	0.325	0.577
		MRT	0.046	0.124	−0.199	0.293	0.695
		STT	−0.120	0.124	−0.372	0.117	0.324
S	SM	MET	1.359	2.916	−4.366	6.992	0.641
		MRT	3.633	2.872	−1.979	9.215	0.202
		STT	−4.992	3.127	−11.151	1.225	0.111
	UT	MET	−0.578	3.248	−6.974	5.739	0.851
		MRT	0.921	2.984	−4.995	6.868	0.758
		STT	−0.343	3.057	−6.367	5.716	0.915
D	SM	MET	0.016	0.031	−0.044	0.076	0.620
		MRT	0.019	0.031	−0.042	0.081	0.521
		STT	−0.036	0.033	−0.102	0.029	0.281
	UT	MET	−0.002	0.034	−0.070	0.065	0.934
		MRT	−0.018	0.033	−0.086	0.046	0.573
		STT	0.021	0.033	−0.045	0.086	0.530
R	SM	MET	0.329	0.268	−0.188	0.859	0.216
		MRT	0.081	0.275	−0.447	0.609	0.777
		STT	−0.411	0.288	−0.984	0.157	0.156
	UT	MET	−0.468	0.303	−1.079	0.124	0.115
		MRT	−0.016	0.294	−0.592	0.575	0.960
		STT	0.485	0.282	−0.067	1.026	0.086
C	SM	MET	0.021	0.031	−0.029	0.073	0.432
		MRT	0.019	0.032	−0.030	0.064	0.483
		STT	−0.041	0.041	−0.087	0.518	0.224
	UT	MET	−0.028	0.022	−0.076	0.026	0.395
		MRT	−0.020	0.019	−0.072	0.032	0.428
		STT	0.049	0.028	−0.002	0.099	0.197

F = Oscillation Frequency; S = Dynamic Stiffness; D = Logarithmic Decrement; R = Mechanical Stress Relaxation Time; C = Ratio of deformation and Relaxation time; SM = superficial masseter muscle; UT = upper trapezius muscle; SD = standard deviation.

**Table 7:** Sex-specific analysis of frequency.

Sex	Muscle	Treatment	Mean	Quantile 0.025	Quantile 0.075	p-value
Female	SM	MET	−0.203	−0.526	0.117	0.999
		MRT	−0.417	−0.771	−0.064	0.189
		STT	−0.083	−0.463	0.284	0.506
	UT	MET	−0.259	−0.675	0.149	0.443
		MRT	0.025	−0.315	0.365	0.177
		STT	−0.274	−0.620	0.069	0.637
Male	SM	MET	−0.369	−0.915	0.168	0.276
		MRT	0.101	−0.404	0.604	0.199
		STT	0.107	−0.525	0.723	0.411
	UT	MET	0.222	−0.371	0.798	0.195
		MRT	−0.400	−0.932	0.131	0.199
		STT	−0.296	−0.826	0.248	0.407

SM = superficial masseter muscle; UT = upper trapezius muscle; MRT = myofascial release technique; MET = muscle energy technique; STT = soft tissue technique; SD = standard deviation. P-value for the simple effects.

### Harms

Participants were instructed to report harms to their physical or mental health to the principal investigator by phone or e–mail, pending their severity. No harms were reported.

## Discussion

To the best of our knowledge, this study is the first to investigate the effect of OMT on the HRMT of healthy subjects. In detail, we used biomechanical and viscoelastic measures to assess the effect of manual techniques with different modalities on muscles with different characteristics. The sample comprised 57 participants (computed: 60; screened: 82; randomised 59) and showed acceptable recruitment and retention rates (72% and 96%, respectively). First, we report significant results for the primary objective. In detail, MET, MRT, and STT applied to the SM and UT (CS was excluded) resulted in significantly decreased muscle tone (F [p = 0.008]), decreased biomechanical (S [p = 0.048] not D [p = 0.527]), and increased viscoelastic properties (R [p < 0.001] and C [p = 0.028]) from baseline to follow-up (Table 5). Notably, decrement (D [p = 0.527]) did not change significantly, but this muscle parameter was previously proposed to be a constant^124^. In contrast, others have reported significant changes of decrement (D) in paraspinal muscles following the application of spinal mobilization in patients with LBP^125^. However, the control group (where participants lay relaxed) showed similar results, and it was hypothesised that the effect may arise due to the relaxed body state^125^. The data suggest that the primary hypothesis can be confirmed, showing that osteopathic techniques modulated the HRMT of treated muscles in healthy subjects by decreasing muscle tone and stiffness while increasing relaxation and creep (decrement did not change significantly). Future studies might assess the effect of OMT on the HRMT of people with MSDs because conditions like LBP and NP are associated with altered muscle properties^109,126,127^. For example, in patients with LBP compared to healthy controls, the lumbar extensor myofascia generally shows increased muscle tone (F), stiffness (S), and decrement (D)^73,126,128,129^ as well as decreased relaxation (R) and creep (C)^69,127,130^. Hence, we propose to examine the effect of OMT on the muscle properties of, for example, the lumbar extensor myofascia or the upper trapezius muscles in patients with LBP or NP, respectively. Based on the present findings, we hypothesise that osteopathic interventions will decrease muscle tone, stiffness, and possibly decrement, and increase relaxation and creep in this population. However, future studies are needed to validate or falsify this hypothesis. Second, we demonstrate no significant results for the secondary objective (using simple effects). However, we detected a non-significant trend (p > 0.05) suggesting that the decrease in muscle tone (F), decrease in biomechanical (D not S), and increase in viscoelastic properties (R and C) were achieved through MET and MRT (not STT) when applied to the SM, and through STT (not MET and MRT) when applied to the UT (Table 6). Notably, this tendency was consistent for all muscle properties except for muscle stiffness (S). In other words, the smaller and thinner muscle (SM) responded (as expected) to the active (MET) and low-pressure technique (MRT), whereas the larger and thicker muscle (UT) responded (as expected) to the high-pressure technique (STT). The reported trend is fairly consistent with the secondary hypothesis (considering that the CS was excluded) but requires further scrutiny for verification or falsification. Lastly, subgroup analysis for the primary objective revealed a significant sex-specific difference for muscle tone (p = 0.030) but no other muscle properties (p > 0.05). Overall, female subjects showed greater descriptive changes (mean values for primary objective) in all muscle properties than male subjects (Table 5). For the secondary objective, no significant sex-specific difference was found but an interesting tendency showed that the muscle tone of the UT increased after MET and decreased after MRT in males, whereas the opposite was reported for females (Table 7 and Figs. 4, 5). The reason for this trend is unclear but we hypothesise, based on clinical experience, that males tend to exaggerate the counterpressure during MET techniques (presumably to demonstrate strength) which may have increased muscle tone.

To date, the mechanisms underlying these reported changes in HRMT following OMT are unclear and require further investigation. There might be biological, psychological, and social factors involved. However, based on the context of this study (comprising a short treatment time, strong manual focus, and unsound therapeutic alliance), we speculate that a biological mechanism of action is most probable. Though it might be the case that the three techniques have the same, different, overlapping, or multiple mechanisms of action, we suggest that mechanotransduction may underlie these changes. In detail, mechanotransduction suggests that extracellular mechanical signals are converted into intracellular chemical signals (and changes in gene expression) via integrins, which physically couple the extracellular matrix (including collagen fibres) with the intracellular cytoskeleton (including actomyosin filaments)^131^. As the HRMT depends on the interaction of cellular actomyosin filaments^53^, we hypothesise that mechanical stimuli provided during osteopathic treatment may change the tensional forces within the (collagen fibres of the) extracellular matrix and, through integrins, within the (actomyosin filaments of the) intracellular cytoskeleton, thereby modifying the HRMT.

Overall, there is a scarcity of studies assessing the effect of manual treatment on muscle properties. Moreover, the available literature is focused on muscle stiffness and tone, whereas decrement, relaxation, and creep are mostly not considered. Therefore, our findings complement the existing body of evidence. Taking these factors into account, our results are largely consistent with the current literature. For example, in healthy participants, it was shown that manual therapy can reduce muscle stiffness^132^; this was demonstrated for MET^133^, MRT (or self-MRT, respectively, which mimics manual MRT with a foam roller)^134^, and STT (or massage, respectively, which resembles STT using deep pressure gliding strokes)^135^. Similarly, it was revealed that trigger points have an increased muscle tone and stiffness^106^, which can be decreased through manual myofascial release^136^. Notably, manual techniques might also reduce muscle tone and stiffness in participants with MSDs^137^. Particularly in patients with LBP, increased paravertebral muscle tone and stiffness were demonstrated^127^, which could be reduced through manual techniques like spinal mobilisation^125^. However, further research is required to substantiate these findings. Also, it is important to note that muscle properties like stiffness do not seem to correlate with pain (in patients with chronic NP and LBP), because muscle stiffness typically returns to baseline one day after treatment (using cupping massage) even if the pain improves^138^. Moreover, we need to consider other factors that could have swayed the results, such as sex, age, and handedness. The sample in this study was relatively homogenous comprising predominantly female (68%), young (22.7 ± 4.5 years), and right-handed (91%) subjects. In general, muscle tone and stiffness are reported to be greater in males than females^72–74^, however, there are also (partially) conflicting findings^70,71,75^. Nonetheless, higher muscle tone and stiffness in males may arise due to differences in muscle size, mass, conditioning, and fiber composition between the sexes^124,139^, which possibly relate to differences in sex hormones^70^ and skinfold thickness^75^. Beyond sex, significant differences in muscle stiffness have also been reported between age groups (higher in elderly and middle-aged than young individuals)^74^ and handedness (higher on the side of the dominant hand)^73^. Although subgroup analysis was performed for sex, we did not consider the impact of age (because only two subjects were over thirty years old) and handedness (because all measures were taken on the right side and only five subjects were left-handed).

There are limitations to consider when interpreting the results of this study. First and foremost, one of the three conditions was dropped because of frequent measurement errors. In detail, all missing values (8.9% of all numbers analysed) originated from myotonometer measurements of the CS (not SM or UT), which was consequently excluded from the analysis. These dependent missing values limited the number of observations available to analysis and therefore reduced the statistical power^140^. Further, dropping one condition increases the likelihood of reporting false-positive results^141^. Presumably, these missing values arose because the feasibility of the MyotonPRO is limited to measurements of muscles that are thicker than 3 mm^68^ and not located near the bone^142,143^ (Box 5).

Another shortfall is that the results are informative of an immediate-term effect (approximately five minutes after treatment). Thus, the measured tissue response may be reflective of the thixotropy effect^144^; albeit the size of this effect remains unclear^145^. Future research may assess if these changes are enduring in the short, medium, and long term. Further, it is noteworthy that participants were assessed in supine position although the HRMT relates to the biotensegrity system and posture^53^. Similarly, it would have been reasonable to measure active muscle tone using EMG. Without it, we cannot verify that participants were fully relaxed. This may have biased the results because active muscle contractions increase muscle tone, stiffness, and elasticity^146^. In the future, myotonometer and EMG measurements may be applied simultaneously (where possible) to ensure that HRMT is measured (i.e., ensuring that active muscle tone is avoided, and passive muscle tone is maintained). Furthermore, a disadvantage of the study procedure was that participants were measured and treated at different times of the day (between 9 a.m. and 4 p.m.). Therefore, time-dependent physiological variations in muscle tone were not considered. This might have affected the results because the resting muscle tone was shown to fluctuate across the day^147^. Other barriers to interpreting the results relate to the interventions. For one, manual techniques are often loosely defined, and it was particularly difficult to find literature describing the same manual procedure as the STT used in this study. In the end, we settled to include research on massage techniques, which employ similar parameters than the STT. Future research might consider not examining manual techniques (e.g., MET, MRT, and STT) but rather their biophysical parameters (e.g., stretch, compression, shear, and torque forces) to ensure optimal comparability^148,149^. Furthermore, the manual techniques were applied for five minutes, which appears brief but seems to be sufficient for one muscle to be treated with one technique (because the therapists perceived palpatory signs of release/relaxation). Also, we cannot rule out that differences between therapists might have influenced the results (e.g., due to sex differences). However, consensus training was implemented before the trial and the therapists exhibited similar characteristics in terms of age and experience. Moreover, the interventions encompassed common manual techniques with different characteristics, however, single manual techniques are not representative of person-centered osteopathic care. Further, subgroup analysis revealed a significant sex-specific difference in F (but no other muscle properties) from baseline to follow-up for the primary objective (but not secondary objective). Thus, the generalisability of this result may be limited to females. However, male subjects were underrepresented in our sample (n = 18; 32%) and future research should recruit sex-balanced samples to substantiate these findings. Lastly, it is unclear if OMT could also have the opposite effect. In detail, it has previously been assumed that OMT restores normal muscle tone where it is altered^36–39^, meaning that high muscle tone decreases, and low muscle tone increases, when OMT is applied^57^. Therefore, future studies might assess the effect of OMT on conditions associated with both hypertonia and hypotonia.

In this study, we demonstrate that OMT modifies the HRMT in healthy participants without significant interaction of muscle-technique pairs. The mechanisms underlying these changes are unclear and the results are limited by the exclusion of one condition. Our findings are largely consistent with previous research but limited by the immediate term measurement. Future studies should modify the protocol and assess if these effects are reproducible (and beneficial) in patients with MSDs. In the end, although speculative, we hypothesise that modifying the HRMT may be a mechanism of action underlying manual techniques.

Box 5 CS characteristics Prior to the trial, we reviewed the literature on the thickness of the CS, which was reported with a mean maximum thickness of 5.50 ± 0.91 mm in healthy subjects^92^. However, because the measurements of the CS were frequently inconsistent, we reviewed the literature again and found other studies accounting for an average thickness of approximately 1.62 ± 0.4 mm^95^, 2–3 mm^150^, and ~ 2.4–2.8 mm^151^, respectively. Thus, we acknowledge a flaw in our initial literature search and suggest that the CS may be too thin and/or near the bone to be consistently measured using the MyotonPRO.

## Conclusion

Taken together, MRT, MET, and STT significantly decreased F and S (not D) and increased R and C of the SM and UT (CS was not measurable) in healthy subjects. Hence, OMT effectively modified the HRMT, and the primary objective can be confirmed. In contrast, the effect of MRT, MET, and STT on the HRMT was not significantly modulated by muscle–technique pairs in healthy subjects. Hence, it is uncertain if some techniques change the HRMT of some muscles more effectively, and the secondary objective must be rejected. Subgroup analysis revealed a significantly greater reduction of F (but no other muscle properties) in female subjects for the primary, but not the secondary, objective. These findings may inform future research investigating the effect of OMT on the HRMT in patients with MSDs.

## Data Availability

The authors confirm that the data supporting the findings of this study are available within the article.

## Abbreviations

MRT: Myofascial release technique
MET: Muscle energy technique
STT: Soft tissue technique
CS: Corrugator supercilii muscle
SM: Superficial masseter muscle
UT: Upper trapezius muscle
F: Oscillation frequency
S: Dynamic stiffness
D: Logarithmic decrement
R: Mechanical stress relaxation time
C: Ratio of deformation and relaxation time
MSDs: Musculoskeletal disorders
HRMT: Human resting muscle tone
OMT: Osteopathic manipulative treatment
EMG: Electromyography
MPs: Measurement points
NP: Neck pain
LBP: Low back pain
